# Closed-Loop Neuroscience and Non-Invasive Brain Stimulation: A Tale of Two Loops

**DOI:** 10.3389/fncel.2016.00092

**Published:** 2016-04-07

**Authors:** Christoph Zrenner, Paolo Belardinelli, Florian Müller-Dahlhaus, Ulf Ziemann

**Affiliations:** Brain Networks and Plasticity Laboratory, Department of Neurology and Stroke and Hertie-Institute for Clinical Brain Research, University of TübingenTübingen, Germany

**Keywords:** closed-loop, EEG, TMS, non-invasive brain stimulation, NIBS

## Abstract

Closed-loop neuroscience is receiving increasing attention with recent technological advances that enable complex feedback loops to be implemented with millisecond resolution on commodity hardware. We summarize emerging conceptual and methodological frameworks that are available to experimenters investigating a “brain in the loop” using non-invasive brain stimulation and briefly review the experimental and therapeutic implications. We take the view that closed-loop neuroscience in fact deals with two conceptually quite different loops: a “brain-state dynamics” loop, used to couple with and modulate the trajectory of neuronal activity patterns, and a “task dynamics” loop, that is the bidirectional motor-sensory interaction between brain and (simulated) environment, and which enables goal-directed behavioral tasks to be incorporated. Both loops need to be considered and combined to realize the full experimental and therapeutic potential of closed-loop neuroscience.

## Introduction

Much has been learned about the mechanics of the brain by treating it as a “black box”, placed in a controlled laboratory environment and stimulated by the experimenter in an open-loop fashion using a pre-defined stimulus protocol to determine its input-output characteristics and how these may be modulated. This approach has been fruitful also in the field of non-invasive brain stimulation (NIBS) and transcranial magnetic stimulation (TMS) in particular, enabling significant advances in the understanding of the functional and pharmacological basis of cortical dynamics (Rothwell et al., 1991; Hallett, 2000; Ziemann et al., 2015).

However, in spite of the significant advances of the past 30 years, TMS has yet to realize its full potential, especially with regard to therapy (Lefaucheur et al., 2014): it is a field plagued with ill-understood intra- and inter-individual variability, effects that hold on average but not reliably for any one individual subject or patient (Hamada et al., 2013; López-Alonso et al., 2014, 2015; Wiethoff et al., 2014; Horvath et al., 2015). One possible origin of the variability in the response to brain stimulation is the variability of instantaneous brain state at the time of stimulation (Ridding and Ziemann, 2010; Li et al., 2015). Recent advances in combining TMS with electroencephalogram (EEG) are enabling us to address this issue by designing stimulation protocols that are controlled by the EEG signal and thus “close the loop” around the brain in a very direct way, short-circuiting the motor-sensory loop (Bergmann et al., 2012).

Moreover, there are compelling conceptual considerations that motivate a “closed-loop” approach: the brain is not a black box; it is not a mere transducer of input to output signals but a generator of behavior—and behavior requires an environment, that is, an entailment between an output of the brain and a (feedback) input to the brain.

In the following, we discuss the conceptual context of closed-loop methods in general, the possibilities and technical challenges of using EEG and TMS to implement a closed-loop set-up, and the implications of this approach to neurophysiology. We review some of the current research and present preliminary technical results from our own lab supporting the expectation that brain-state dependent brain-stimulation will be significantly more effective at modulating neural pathways than current open-loop protocols.

## Closed-Loop Method

### Conceptual Considerations

**Figure 1:**
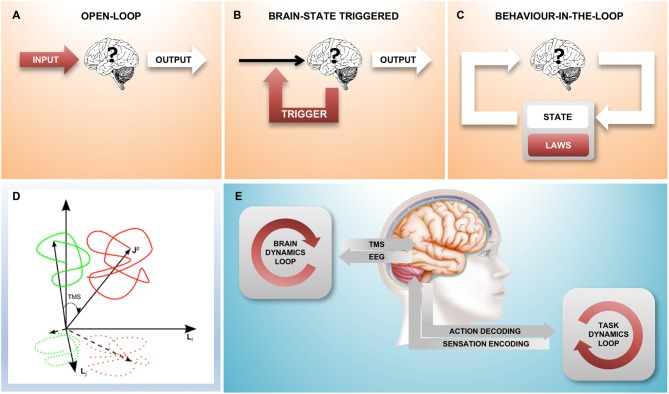
**A family of different closed-loop designs to couple with brain state dynamics. (A)** Traditional “black box” experiment where there is no environment that the brain can act on: the stimulation is predetermined by the experimenter, the experimental observable is the output from the brain. **(B)** The feedback loop triggers a stimulus based on a spontaneously occurring instantaneous brain state; here, the environment is static and without state. **(C)** This loop represents an agent-environment interaction where the environment has its own internal dynamics, consisting of state (which is fully observable) and the equations of motion (the laws that govern the behavior of the environment). The combined result is an interacting complex system. Areas shaded red indicate parts of the set-up under experimental control, white areas show the “observable behavior”. **(D)** Shows the relationship between “true” brain state trajectories and the projection onto the measured electroencephalogram (EEG) signal as well as the effect that a transcranial magnetic stimulation (TMS) pulse has shifting state to a new position (figure taken from Mutanen et al., 2013) **(E)**. An experimental closed-loop EEG-TMS set-up configured to couple with cortical dynamics during a simultaneously executed motor-sensory task. Two conceptually different feedback loops can be distinguished, a “brain-state dynamics” loop that is designed to influence the trajectory of the brain state and a “task dynamics” loop that enables active interaction with a (real or computer simulated) environment through the motor and sensory system and the respective encoding stages.

An experiment may be considered a “closed-loop” experiment when actions (output from the brain) have consequences (future input to the brain). This is the natural state of affairs for an organism roaming through the environment; indeed, the output of the brain is relevant *only* insomuch as it has the ability to impact the future and hence the input the brain receives. However, this is typically not the case in the neurophysiology lab: in a controlled “black-box” experiment, the input to the brain is a stimulus protocol arbitrarily defined by the experimenter and the output from the brain is the experimenter’s relevant measure, the dependent variable. In such an “open-loop” experiment, it is only the input to the brain that has a consequence (Figure 1A), the output has no effect on the future and, from the point of view of the brain, is therefore wholly irrelevant.

It is of course possible to “close the loop” in the laboratory by establishing a causal relationship between the measured output of the brain and the stimulus generator. Indeed, there are two quite distinct ways in which this can be done: In the simpler case, the stimulus is applied as a function of the simultaneously measured instantaneous brain state (Figure 1B). In this scenario, the neuronal “output” of the brain influences the input to the brain and in this sense the loop is closed; however, the loop is “stateless” and the measured activity of the brain fully determines the input stimulus at any given instant. This kind of a loop does not establish a dynamically changing “outside environment”, there is nothing outside of the brain that has any influence on the input signal.

The more complex closed-loop scenario (Figure 1C) is that of a nervous system experimentally coupled with a physical or simulated outside environment that, similarly to the brain, has its own dynamically changing state and a set of laws that define the evolution of the system (the laws of physics). Here, as in the interaction with the real-world through the motor and sensory systems, neither the brain, nor the environment (and the other brains within it) individually determine the time-course of the stimulus; the input to the brain arises from the complex interactions within the system, which results in the “behavior-in-the-loop” of an “embodied” system.

This third scenario is qualitatively different from the second scenario in that any causal explanation of the dynamic interaction between brain and environment can no longer be limited to the state and transition dynamics of the brain alone, but must take into account the state of the environment, too. Indeed, whereas the complete state of the brain is necessarily not accessible by the experimenter, we can present the subject with a simple constructed or simulated environment to which we do have comprehensive access and which can be experimentally controlled.

This is also the key difference to the ethological approach of just observing an animal in its natural environment: in the fully closed-loop setting as described above, the experimenter is able to access and interfere with the flow of information between neural system and environment on the one hand and the state and transition dynamics of the environment on the other. Instead of observing the output in response to different inputs, the “behavior” of the neural system now includes both the brain and the environment and can be studied in environments following different equations of motion (state transition laws). Whereas in the open-loop stimulus-response setting, the scope of what may be experimentally observed is limited to a single variable (the “response”), in the closed-loop setting the possible state space trajectories that the neural system may take in conjunction with the environment are not so constrained.

There is large body of work creating an artificial connection between the brain and the natural environment through a brain-computer interface (Buch et al., 2008; Bundy et al., 2012; Ramos-Murguialday et al., 2013), bypassing the (damaged) motor system and receiving proprioceptive and visual feedback through the intact sensory system. However, with the ability to stimulate the brain directly using TMS, the environment that is coupled to the neural system can be a simulation running on a computer. By providing “environment-like” closed-loop feedback protocols, a bi-directionally coupled device can implement therapeutic stimulation paradigms on a microchip that are designed to remodel dysfunctional networks by providing the kind of output-input relationships that the network would experience during “natural” interactions with the environment.

### Closed-Loop Neuroscience Through EEG-Triggered TMS

The insight that a closed-loop experimental approach may enable us to learn new things about the brain that we cannot learn from a “black box” approach is of course not a new one, as both experimental work *in vitro* (Huerta and Lisman, 1993, 1995) and *in vivo* (Siegle and Wilson, 2014) as well as theoretical results (Friston, 2010) attest to. Here we wish to draw attention to the unique potential of non-invasive closed-loop brain stimulation, enabled by the combination of EEG and TMS (Ilmoniemi et al., 1997; Ilmoniemi and Kicić, 2010), and the recent availability of low-cost real-time processor solutions.

TMS is an old technique but it remains the only way to non-invasively excite a specific population of cortical neurons with a spatial resolution of millimeters and a temporal resolution of microseconds (Barker et al., 1985; Hallett, 2000; Müller-Dahlhaus and Vlachos, 2013). Considering the EEG signal conceptually as a lower-dimensional projection of instantaneous brain state, application of a TMS pulse may be viewed as a vector that shifts a spontaneously occurring brain state to a new trajectory (Mutanen et al., 2013, Figure 1D). Importantly, the new state that is reached by the TMS pulse depends on the precise state at the time of stimulation and this is what motivates the development of closed-loop brain-state dependent stimulation paradigms.

The “true” brain state is not accessible, it would constitute a long vector describing the state and activity of each nerve cell and synapse; in practice, the dimensionality is already reduced (information is lost) by the EEG or MEG recording and then further as this is projected onto some quantifiable correlate of a physiological relevant process. In the following description, we focus on what is perhaps the most salient feature of brain state as measured by EEG, i.e., spontaneous oscillatory activity of neuronal populations (Buzsáki and Draguhn, 2004), which takes place on different spatial and temporal scales and can be quantified by several measures, in particular phase.

Spatially, brain states can be observed locally, i.e., in the activity of a specific brain area (Gharabaghi et al., 2014) or in the ensemble activity across large-scale brain networks (Bundy et al., 2012). Temporally, the state of interest can be determined by a certain phase-state of an oscillation cycle (with a timescale of milliseconds) or by changes in spectral power in a specific frequency band (e.g., event-related desynchronization (ERD)), which has a slower time-scale of seconds. The latter has been successfully utilized for BMI-based robot-assisted motor tasks in stroke patients, both in the alpha spectrum of 8–12 Hz (Pfurtscheller and Neuper, 2009) and in the beta spectrum of 16–22 Hz (Kraus et al., 2016).

**Figure 2:**
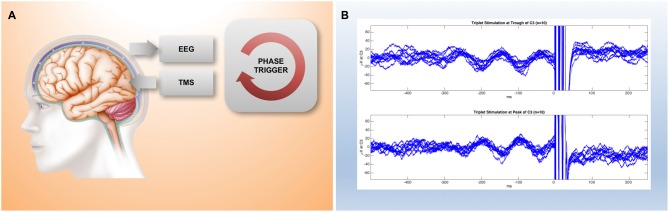
**Preliminary results from a millisecond latency EEG-TMS set-up. (A)** Simplified implementation of a closed-loop brain-state dependent brain-stimulation set-up consisting of EEG stage, real-time digital signal processing stage, and a triggered stimulation stage. **(B)** Raw EEG traces recorded from electrode C3 in the period before a TMS 100 Hz triplet pulse that is triggered by a real-time system based on the preceding 300 ms of EEG data. The system can be configured to trigger the stimulation either at the trough (top trace) or at the peak (bottom trace) of spontaneous alpha activity recorded by EEG over motor cortex.

Closing the loop not only on spectral power but also on instantaneous phase is methodologically more challenging because it requires a real-time signal processing stage with a time-resolution of milliseconds, however, this has recently become possible (see the next section and Figure 2). Finally, in order to go beyond brain states that occur at rest, but instead correspond to a particular cognitive behavioral process, in addition to the “brain dynamics feedback loop”, a “task dynamics” loop will be needed (Figure 1E). EEG-TMS set-ups are already enabling a coupling with cortical dynamics during the performance of a behavioral task in awake human subjects (Thabit et al., 2010; Kraus et al., 2016), and performance dependent feedback has also been shown to support neurorehabilitation in stroke patients (Buetefisch et al., 2011; Ramos-Murguialday et al., 2013).

### Technical Challenges

The implementation of a closed-loop set-up requires several different stages: a brain output measurement stage, a signal processing stage, and a stimulus modulation stage (Figure 2A). This has become feasible by recent advances in information technology, which enable complex computations to be performed in real-time even with low-cost standard hardware.

The performance of the closed-loop system can be characterized by the fundamental sample-time of the processor, the bandwidth of data acquired, the complexity of computations that can be performed within a single time-step, the overall feedback latency through the loop from signal to stimulus, as well as any jitter in that latency. Importantly, a loop latency and jitter in the order of milliseconds is required for phase-dependent stimulation of endogenous brain activity in higher frequency bands (beta, gamma).

Sources of latency include the data processing and transfer buffers at each stage, but also the phase shift inherent to signal filters. Generic general real-time signal processing systems (such as Mathworks Simulink Real-Time or National Instruments LabView) can easily process data in sample time steps <1 ms (Zrenner et al., 2010), however, a specialized biosignal recording device is required that makes a non-buffered stream of data available with a small fixed delay of a few milliseconds.

Until recently, artifacts introduced by the TMS pulse in the simultaneously recorded EEG signal were a major obstacle to closed-loop EEG-TMS because of amplifier saturation. However, with the availability of high dynamic range 24 bit analog-to-digital converters, the stimulus artifact is simply captured by the amplifier and the complexity and discontinuities introduced by previously required sample-and-hold or blanking circuits is obviated. In combination with TMS compatible sintered ring electrodes the TMS artifact duration can be reduced to <10 ms (Virtanen et al., 1999; Bonato et al., 2006; Litvak et al., 2007; Veniero et al., 2009). This leaves muscle artifacts and somatosensory as well as auditory evoked potentials that overlap with the TMS-evoked potentials; the different components are then typically segregated using independent component analysis (ICA).

The most significant limitation concerns real-time analysis of the biosignal data, as it is streamed to the real-time processor. Only a sliding window of data preceding the current time point can be considered and this means that any kind of filtering will cause phase shifts or edge effects that need to be compensated for. Furthermore, since we are only considering an individual epoch in each trial, none of the standard methods for averaged data with a window extending both directions around the event of interest are available.

## Implications of Closed-Loop Approaches for Neurophysiology

Closed-loop non-invasive brain stimulation with millisecond precision enables selective interference with ongoing brain activity and thereby can help to clarify important open questions in neurophysiology: How does the TMS pulse evoke cortico-spinal activity (Rossini et al., 2015)? Which oscillatory brain activity serves which functional role? Such a functional dissection of brain networks “by instantaneous state” adds an additional dimension along which the *in vivo* system can be separated into functional subsystems, similar to what has been achieved by pharmacological (Ziemann et al., 2015) and optogenetic interventions (for a review, see Grosenick et al., 2015).

A pioneering application of closed-loop brain-state triggered stimulation in a “behavior-in-the-loop” setting can be found in an animal experiment by Siegle and Wilson (2014) where freely behaving mice received a stimulus that was triggered by the phase of ongoing hippocampal theta oscillations during the performance of a memory task. Using this closed-loop design the authors could dissect both the role of phase and task segment (encoding vs. retrieval) for task-dependent information processing.

Nevertheless, the implicit physical environment of a freely behaving animal is difficult to capture, the sensory and motor interaction difficult to quantify, and the experimental control and opportunities for intervention limited; we therefore expect that an explicit “task dynamics” loop, in the form of an environment simulated on a computer, will be an increasingly relevant feature of closed-loop designs. Neuronal cell cultures have long been studied as an experimental system with such a view in mind (Shahaf and Marom, 2001; Chao et al., 2008; Keren and Marom, 2014; Potter et al., 2014) and the role of the agent-environment interaction (“relational dynamics”) has also been explored in human behavioral experiments (Marom and Wallach, 2011). These results provide suggestive avenues for the design of closed-loop non-invasive brain stimulation experiments to better understand the brain networks underlying the generation of behavior in human subjects.

## Implications of Closed-Loop Approaches for Therapy

With regard to the therapy of neurological disorders due to “network dysregulation”, closed-loop paradigms can be applied in two different ways: firstly, a control-system theory approach uses the feedback stimulus as a “regulator” to re-“set” the local excitability and activity of a network (see Wallach, 2014 for an *in vitro* example). Secondly, a neuromodulatory approach uses a stimulus train to induce specific long-term plastic changes and thereby re-“wire” the connectivity of the network.

For instance, in a rodent model of generalized epilepsy closed-loop transcranial electrical stimulation based on a threshold mechanism of cortical local field potentials and unitary activity was shown to reduce epileptiform spike-and-wave episodes (Berényi et al., 2012). In humans, closed-loop deep brain stimulation (DBS) paradigms have recently been implemented for the treatment of advanced Parkinson’s disease by applying adaptive DBS whenever recording of local field potentials directly via the DBS electrodes exceeded a threshold in beta power, since exaggerated beta oscillations have been identified as a marker of akinesia and rigidity (Little and Brown, 2012; Little et al., 2013). Adaptive DBS was more effective than conventional open-loop DBS in improving Parkinsonian symptoms, at more than 50% reduction of stimulation time (Little et al., 2013). Likewise, surface EEG ERD over the motor cortex has been used to control DBS for the treatment of essential tremor (Herron et al., 2015).

Another important example for closed-loop stimulation is auditory stimulation during <1 Hz slow-wave sleep, a phase of sleep that is critical to declarative memory consolidation. It was demonstrated in healthy sleeping humans that auditory stimulation in phase with the ongoing rhythmic occurrence of slow oscillation up states measured in the EEG significantly enhanced the slow oscillation rhythm, phase-coupled sleep spindle activity, and in turn, the consolidation of declarative memory (Ngo et al., 2013). Stimulation out of phase with the ongoing slow oscillation rhythm remained ineffective with respect to all readouts.

In summary, EEG brain-state triggered NIBS or behavior-in-the-loop set-ups will enable physicians to interfere with their patients’ ongoing brain activity with high temporal, spatial and spectral precision. NIBS or behavioral neurofeedback can thus be coupled to endogenous brain activity in functionally defined brain networks in real time. This approach has several important advantages. Firstly, neuromodulation can be personalized to individual network function, that is, inter-individual differences in the excitability and connectivity of brain networks can be taken into account. Secondly, the time-course of dynamic changes during network reorganization such as during stroke rehabilitation (Grefkes and Ward, 2013) can be taken into account. Thirdly, since the modifiability of neurons and networks is a function of their recent activity (metaplasticity), which critically determines direction, extent and duration of plasticity in neural networks (Müller-Dahlhaus and Ziemann, 2015), and which can be used to time the stimulation appropriately using a closed-loop method. Implementation of closed-loop neuromodulation in a therapeutic setting will thus critically depend on characterization of informative EEG or MEG parameters for network function and plasticity.

Finally, behavior-in-the-loop set-ups can additionally capitalize on agency and subjectivity of brain-environment interactions. For instance, NIBS can be applied through the “brain dynamics loop” in synchrony to motor-sensory feedback from the “task dynamics loop” (see Figure 1E), reinforcing or interfering with the brain’s wired input-output processing in an agent-environment interaction. In this context, methodological advances of latest closed-loops systems as described above, i.e., low latency and low jitter, will prove essential to match the multiple natural time-scales of the environment that the brain is evolutionary tuned for. It appears promising to further develop this type of comprehensive combined closed-loop intervention in the treatment of neurological and psychiatric diseases.

## Conclusion

Two different closed-loop interactions can be differentiated: a direct coupling to instantaneous brain states through non-invasive brain stimulation (“brain dynamics” loop), and a coupling to an environmental system that presents the brain with the opportunity to generate goal-directed behavior through the motor-sensory loop (“task dynamics” loop). These two approaches to closed-loop neuroscience are conceptually quite different but they are complementary in that they serve to induce and then interfere with specific brain states. Our assertion is that there is significant experimental and therapeutic potential in the application of brain-state dependent brain stimulation while the subject or patient is simultaneously performing a task, where both loops are interactively optimized for neuromodulatory efficacy.

## Author Contributions

All authors listed, have made substantial, direct and intellectual contribution to the work, and approved it for publication.

## Conflict of Interest Statement

The authors declare that the research was conducted in the absence of any commercial or financial relationships that could be construed as a potential conflict of interest.

## References

[B1] Barker A. T. Jalinous R. Freeston I. L. (1985). Non-invasive magnetic stimulation of human motor cortex. Lancet 325, 1106–1107. 10.1016/s0140-6736(85)92413-4 2860322

[B2] Berényi A. Belluscio M. Mao D. Buzsáki G. (2012). Closed-loop control of epilepsy by transcranial electrical stimulation. Science 337, 735–737. 10.1126/science.1223154 22879515 PMC4908579

[B3] Bergmann T. O. Mölle M. Schmidt M. A. Lindner C. Marshall L. Born J.. (2012). EEG-guided transcranial magnetic stimulation reveals rapid shifts in motor cortical excitability during the human sleep slow oscillation. J. Neurosci. 32, 243–253. 10.1523/JNEUROSCI.4792-11.2012 22219286 PMC6621327

[B4] Bonato C. Miniussi C. Rossini P. M. (2006). Transcranial magnetic stimulation and cortical evoked potentials: a TMS/EEG co-registration study. Clin. Neurophysiol. 117, 1699–1707. 10.1016/j.clinph.2006.05.006 16797232

[B5] Buch E. Weber C. Cohen L. G. Braun C. Dimyan M. A. Ard T.. (2008). Think to move: a neuromagnetic brain-computer interface (BCI) system for chronic stroke. Stroke 39, 910–917. 10.1161/STROKEAHA.107.505313 18258825 PMC5494966

[B6] Buetefisch C. Heger R. Schicks W. Seitz R. Netz J. (2011). Hebbian-type stimulation during robot-assisted training in patients with stroke. Neurorehabil. Neural Repair 25, 645–655. 10.1177/1545968311402507 21606211

[B7] Bundy D. T. Wronkiewicz M. Sharma M. Moran D. W. Corbetta M. Leuthardt E. C. (2012). Using ipsilateral motor signals in the unaffected cerebral hemisphere as a signal platform for brain-computer interfaces in hemiplegic stroke survivors. J. Neural Eng. 9:036011. 10.1088/1741-2560/9/3/036011 22614631 PMC3402181

[B8] Buzsáki G. Draguhn A. (2004). Neuronal oscillations in cortical networks. Science 304, 1926–1929. 10.1126/science.1099745 15218136

[B9] Chao Z. C. Bakkum D. J. Potter S. M. (2008). Shaping embodied neural networks for adaptive goal-directed behavior. PLoS Comput. Biol. 4:e1000042. 10.1371/journal.pcbi.1000042 18369432 PMC2265558

[B10] Friston K. (2010). The free-energy principle: a unified brain theory? Nat. Rev. Neurosci. 11, 127–138. 10.1038/nrn2787 20068583

[B11] Gharabaghi A. Kraus D. Leão M. T. Spüler M. Walter A. Bogdan M.. (2014). Coupling brain-machine interfaces with cortical stimulation for brain-state dependent stimulation: enhancing motor cortex excitability for neurorehabilitation. Front. Hum. Neurosci. 8:122. 10.3389/fnhum.2014.00122 24634650 PMC3942791

[B12] Grefkes C. Ward N. S. (2013). Cortical reorganization after stroke how much and how functional? Neuroscientist 20, 56–70. 10.1177/1073858413491147 23774218

[B13] Grosenick L. Marshel J. H. Deisseroth K. (2015). Closed-loop and activity-guided optogenetic control. Neuron 86, 106–139. 10.1016/j.neuron.2015.03.034 25856490 PMC4775736

[B15] Hallett M. (2000). Transcranial magnetic stimulation and the human brain. Nature 406, 147–150. 10.1038/35018000 10910346

[B16] Hamada M. Murase N. Hasan A. Balaratnam M. Rothwell J. C. (2013). The role of interneuron networks in driving human motor cortical plasticity. Cereb. Cortex 23, 1593–1605. 10.1093/cercor/bhs147 22661405

[B17] Herron J. Denison T. Chizeck H. J. (2015). “Closed-loop DBS with movement intention,” in 7th Annual International IEEE EMBS Conference on Neural Engineering (NER), Montpellier, France.

[B18] Horvath J. C. Forte J. D. Carter O. (2015). Evidence that transcranial direct current stimulation (tDCS) generates little-to-no reliable neurophysiologic effect beyond MEP amplitude modulation in healthy human subjects: a systematic review. Neuropsychologia 66C, 213–236. 10.1016/j.neuropsychologia.2014.11.021 25448853

[B19] Huerta P. T. Lisman J. E. (1993). Heightened synaptic plasticity of hippocampal CA1 neurons during a cholinergically induced rhythmic state. Nature 364, 723–725. 10.1038/364723a0 8355787

[B20] Huerta P. T. Lisman J. E. (1995). Bidirectional synaptic plasticity induced by a single burst during cholinergic theta oscillation in CA1 *in vitro*. Neuron 15, 1053–1063. 10.1016/0896-6273(95)90094-2 7576649

[B21] Ilmoniemi R. J. Kicić D. (2010). Methodology for combined TMS and EEG. Brain Topogr. 22, 233–248. 10.1007/s10548-009-0123-4 20012350 PMC2800178

[B22] Ilmoniemi R. J. Virtanen J. Ruohonen J. Karhu J. Aronen H. J. Näätänen R.. (1997). Neuronal responses to magnetic stimulation reveal cortical reactivity and connectivity. Neuroreport 8, 3537–3540. 10.1097/00001756-199711100-00024 9427322

[B23] Keren H. Marom S. (2014). Controlling neural network responsiveness: tradeoffs and constraints. Front. Neuroeng. 7:11. 10.3389/fneng.2014.00011 24808860 PMC4010759

[B24] Kraus D. Naros G. Bauer R. Leão M. T. Ziemann U. Gharabaghi A. (2016). Brain-robot interface driven plasticity: distributed modulation of corticospinal excitability. Neuroimage 125, 522–532. 10.1016/j.neuroimage.2015.09.074 26505298

[B25] Lefaucheur J. P. André-Obadia N. Antal A. Ayache S. S. Baeken C. Benninger D. H.. (2014). Evidence-based guidelines on the therapeutic use of repetitive transcranial magnetic stimulation (rTMS). Clin. Neurophysiol. 125, 2150–2206. 10.1016/j.clinph.2014.05.021 25034472

[B26] Li L. M. Uehara K. Hanakawa T. (2015). The contribution of interindividual factors to variability of response in transcranial direct current stimulation studies. Front. Cell. Neurosci. 9:181. 10.3389/fncel.2015.00181 26029052 PMC4428123

[B27] Little S. Brown P. (2012). What brain signals are suitable for feedback control of deep brain stimulation in Parkinson’s disease? Ann. N Y Acad. Sci. 1265, 9–24. 10.1111/j.1749-6632.2012.06650.x 22830645 PMC3495297

[B28] Little S. Pogosyan A. Neal S. Zavala B. Zrinzo L. Hariz M.. (2013). Adaptive deep brain stimulation in advanced Parkinson’s disease. Ann. Neurol. 74, 449–457. 10.1002/ana.23951 23852650 PMC3886292

[B29] Litvak V. Komssi S. Scherg M. Hoechstetter K. Classen J. Zaaroor M.. (2007). Artifact correction and source analysis of early electroencephalographic responses evoked by transcranial magnetic stimulation over primary motor cortex. Neuroimage 37, 56–70. 10.1016/j.neuroimage.2007.05.015 17574872

[B30] López-Alonso V. Cheeran B. Río-Rodríguez D. Fernández-Del-Olmo M. (2014). Inter-individual variability in response to non-invasive brain stimulation paradigms. Brain Stimul. 7, 372–380. 10.1016/j.brs.2014.02.004 24630849

[B31] López-Alonso V. Fernández-Del-Olmo M. Costantini A. Gonzalez-Henriquez J. J. Cheeran B. (2015). Intra-individual variability in the response to anodal transcranial direct current stimulation. Clin. Neurophysiol. 126, 2342–2347. 10.1016/j.clinph.2015.03.022 25922127

[B32] Marom S. Wallach A. (2011). Relational dynamics in perception: impacts on trial-to-trial variation. Front. Comput. Neurosci. 5:16. 10.3389/fncom.2011.00016 21647414 PMC3103212

[B33] Müller-Dahlhaus F. Vlachos A. (2013). Unraveling the cellular and molecular mechanisms of repetitive magnetic stimulation. Front. Mol. Neurosci. 6:50. 10.3389/fnmol.2013.00050 24381540 PMC3865432

[B34] Müller-Dahlhaus F. Ziemann U. (2015). Metaplasticity in human cortex. Neuroscientist 21, 185–202. 10.1177/1073858414526645 24620008

[B35] Mutanen T. Nieminen J. O. Ilmoniemi R. J. (2013). TMS-evoked changes in brain-state dynamics quantified by using EEG data. Front. Hum. Neurosci. 7:155. 10.3389/fnhum.2013.00155 23630486 PMC3635036

[B36] Ngo H.-V. V. Martinetz T. Born J. Mölle M. (2013). Auditory closed-loop stimulation of the sleep slow oscillation enhances memory. Neuron 78, 545–553. 10.1016/j.neuron.2013.03.006 23583623

[B37] Pfurtscheller G. Neuper C. (2009). “Dynamics of sensorimotor oscillations in a motor task,” in Brain-Computer Interfaces, eds Graimann B. Pfurtscheller G. Allison B. (Springer: Springer Berlin Heidelberg), 47–64.

[B38] Potter S. M. El Hady A. Fetz E. E. (2014). Closed-loop neuroscience and neuroengineering. Front. Neural Circuits 8:115. 10.3389/fncir.2014.00115 25294988 PMC4171982

[B40] Ramos-Murguialday A. Broetz D. Rea M. Läer L. Yilmaz Ö. Brasil F. L.. (2013). Brain-machine interface in chronic stroke rehabilitation: a controlled study. Ann. Neurol. 74, 100–108. 10.1002/ana.23879 23494615 PMC3700597

[B41] Ridding M. C. Ziemann U. (2010). Determinants of the induction of cortical plasticity by non-invasive brain stimulation in healthy subjects. J. Physiol. 588, 2291–2304. 10.1113/jphysiol.2010.190314 20478978 PMC2915507

[B42] Rossini P. Burke D. Chen R. Cohen L. Daskalakis Z. Di Iorio R.. (2015). Non-invasive electrical and magnetic stimulation of the brain, spinal cord, roots and peripheral nerves: basic principles and procedures for routine clinical and research application. An updated report from an IFCN Committee. Clin. Neurophysiol. 126, 1071–1107. 10.1016/j.clinph.2015.02.001 25797650 PMC6350257

[B43] Rothwell J. C. Thompson P. D. Day B. L. Boyd S. Marsden C. D. (1991). Stimulation of the human motor cortex through the scalp. Exp. Physiol. 76, 159–200. 10.1113/expphysiol.1991.sp003485 2059424

[B44] Shahaf G. Marom S. (2001). Learning in networks of cortical neurons. J. Neurosci. 21, 8782–8788. 11698590 10.1523/JNEUROSCI.21-22-08782.2001 PMC6762268

[B45] Siegle J. H. Wilson M. A. (2014). Enhancement of encoding and retrieval functions through theta phase-specific manipulation of hippocampus. Elife 3:e03061. 10.7554/elife.03061 25073927 PMC4384761

[B46] Thabit M. N. Ueki Y. Koganemaru S. Fawi G. Fukuyama H. Mima T. (2010). Movement-related cortical stimulation can induce human motor plasticity. J. Neurosci. 30, 11529–11536. 10.1523/JNEUROSCI.1829-10.2010 20739575 PMC6633334

[B47] Veniero D. Bortoletto M. Miniussi C. (2009). TMS-EEG co-registration: on TMS-induced artifact. Clin. Neurophysiol. 120, 1392–1399. 10.1016/j.clinph.2009.04.023 19535291

[B48] Virtanen J. Ruohonen J. Näätänen R. Ilmoniemi R. J. (1999). Instrumentation for the measurement of electric brain responses to transcranial magnetic stimulation. Med. Biol. Eng. Comput. 37, 322–326. 10.1007/bf02513307 10505382

[B49] Wallach A. (2014). The response clamp: functional characterization of neural systems using closed-loop control. Front. Neural Circuits 7:5. 10.3389/fncir.2013.00005 23382712 PMC3558724

[B50] Wiethoff S. Hamada M. Rothwell J. C. (2014). Variability in response to transcranial direct current stimulation of the motor cortex. Brain Stimul. 7, 468–475. 10.1016/j.brs.2014.02.003 24630848

[B52] Ziemann U. Reis J. Schwenkreis P. Rosanova M. Strafella A. Badawy R.. (2015). TMS and drugs revisited 2014. Clin. Neurophysiol. 126, 1847–1868. 10.1016/j.clinph.2014.08.028 25534482

[B53] Zrenner C. Eytan D. Wallach A. Thier P. Marom S. (2010). A generic framework for real-time multi-channel neuronal signal analysis, telemetry control and sub-millisecond latency feedback generation. Front. Neurosci. 4:173. 10.3389/fnins.2010.00173 21060803 PMC2972682

